# Clinical Implications of Herbal Supplements in Conventional Medical Practice: A US Perspective

**DOI:** 10.7759/cureus.26893

**Published:** 2022-07-15

**Authors:** Gashaw Hassen, Gizeshwork Belete, Keila G Carrera, Rosemary O Iriowen, Haimanot Araya, Tadesse Alemu, Nebiyou Solomon, Diwas S Bam, Sophia M Nicola, Michael E Araya, Tadesse Debele, Michlene Zouetr, Nidhi Jain

**Affiliations:** 1 Internal Medicine, University of Maryland Capital Region Medical Center, Largo, USA; 2 Medicine, Addis Ababa University, Addis Ababa, ETH; 3 Progressive Care, Mercy Medical Center, Baltimore, USA; 4 Medicine and Surgery, Parma University, Parma, ITA; 5 Internal Medicine, Saint Agnes Hospital, Baltimore, USA; 6 Gastroenterology, Universidad de Oriente (VEN), Maturin, VEN; 7 Public Health, Thomas Jefferson University, Philadelphia, USA; 8 General Practice, Jimma University, Jimma, ETH; 9 Internal Medicine, Dirgh Jeevan Health Care and Research Center, Kathmandu, NPL; 10 Rheumatology, Howard University Hospital, Washington, DC, USA; 11 Internal Medicine, Jimma University, Jimma, ETH; 12 Internal Medicine, Northside Gwinnett Hospital, Lawrenceville, USA; 13 Family Medicine, Midwestern University Arizona College of Osteopathic Medicine, Kingman, USA; 14 Family Medicine, American Institute of Antigua College of Medicine, St. John's, ATG; 15 Medicine and Surgery, Himalayan Institute of Medical Sciences, Dehradun, IND; 16 Hematology and Oncology, Brooklyn Cancer Care, Brooklyn, USA; 17 Internal Medicine, Sir Ganga Ram Hospital, New Delhi, IND

**Keywords:** safety and efficacy, fda-food and drug administration, herb-drug interactions (hdi), phytotherapy, herbal remedies, herbal medicines, herbal products, herbal supplements, complementary and alternative medicine(cam), conventional medicine (cm)

## Abstract

Herbal supplements are common complementary and alternative medicine (CAM) approaches with an ever-increasing use trend in the last two decades among the US population. Self-medication with herbal supplements which are promoted for general well-being, weight loss, immunity, and memory boost, and mental and physical health claims are very prevalent. There is a misperception that herbal supplements are harmless as they are prepared from natural sources. Unlike conventional drugs, the US Food and Drug Administration (FDA) does not regulate herbal supplements for premarketing purity and potency. Hence, there is a growing concern for health risks due to misbranded toxic ingredients, contaminants, adulterants, and herb-drug interactions (HDI) with co-administered drugs. The spectrum of pharmacological and toxicological effects of herbal supplements includes deranged lab results, allergic reactions, genotoxicity, carcinogenicity, teratogenicity, organ damage, and even fatality contributing to sizable emergency visits and hospitalizations in the US. The use of herbal supplements should be carefully monitored in high-risk groups such as pediatric and geriatric populations, pregnant women, breastfeeding mothers, immunocompromised patients, and surgical candidates. The deleterious health effects of herbal supplements are loosely addressed in conventional medical practice in part due to the limited knowledge of practitioners. This comprehensive narrative review aims to explore the clinical implications of herbal supplements in order to fill the knowledge gaps by summarizing scientific publications. It also highlights the pivotal roles physicians can play in minimizing the health risks of herbal supplements by encouraging patients to disclose usage through a non-judgmental approach, employing HDI screening tools, and reporting adverse reactions to FDA. Formal training of physicians, a standardized pharmacovigilance system, stricter regulation of the herbal industry, and more scientific studies are keys to establishing herbal safety and efficacy in clinical practice.

## Introduction and background

Despite the predominance of conventional medicine (CM) in modern American medical practice which relies on drugs, surgery, and radiation, alternate and competitive healing traditions categorized under complementary and alternative medicine (CAM) is flourishing and becoming increasingly prevalent [1-3]. Complementary medicine is a non-mainstream approach used together with CM whereas alternative medicine is termed when the non-mainstream approach is used in place of CM [4]. There are five major categories of CAM: (1) Mind-body interventions (meditation, hypnosis, yoga); (2) Biologically based treatments (herbs, probiotics, vitamins, minerals); (3) Manipulative and body-based methods (massage, osteopathic manipulation, chiropractic manipulation); (4) Energy Therapies (spiritual healing, Qi do, distant healing); and (5) Alternative medical systems (naturopathy, homeopathy, Ayurveda, Traditional Chinese medicine) [5-6].

CM is also referred to as western medicine, allopathic medicine, biomedicine, mainstream medicine, orthodox medicine, or standard medicine [1]. Despite the popularity of CAM over the recent decades, there exists knowledge and a communication gap among health care professionals with respect to counseling patients about efficacy, safety, and drug interactions attributed to a lack of evidence-based information and formal training. Whereas, some academic institutions are recognizing and incorporating CAM into medical education, clinical practice, and research [7].

A study found that around 42% of the US population used at least one CAM therapy but less than 40% of them disclosed their use to a physician [5]. According to the 2007 National Health Interview Survey (NHIS) conducted by the Centers for Disease Control and Prevention's (CDC) National Center for Health Statistics (NCHS), 40% of adults and 12% of children in the US used CAM [8]. Total visits to CAM providers are exceeding that of primary-care physicians (PCPs) with billions of out-of-the-pocket spending [5]. Surprisingly, CAM is practiced by all age groups across all the US states [9].

The most commonly used CAM approaches among US adults were non-vitamin non-mineral dietary supplements (17.9%), chiropractic or osteopathic manipulation (8.5%), yoga (8.4%), massage (6.8%), and meditation (4.1%) [10]. Natural products (3.9%), chiropractic and osteopathic manipulation (2.8%), deep breathing (2.2%), yoga (2.1%), and homeopathic treatment (1.3%) were the top five CAM therapies among US children [9]. Health conditions for which CAM was frequently used in adults include pain syndrome (28.2%), arthritis (3.5%), neuropsychiatric conditions (5.8%), and cholesterol (2.1%) [9]. Children were provided CAM therapies for back pain (16.8%), head or chest cold (9.5%), anxiety or depression (4.5%), stomach upset (3.7%), severe headache or migraine (3.1%), insomnia (2.2%) [9].

Herbal supplements are the most widely used CAM with an increasing trend particularly over the last two decades [11]. In 1997, an estimated 15 million adults took herbal remedies concurrently with prescription medications which led to negative health consequences [5]. Despite a few herbal supplements with evidence of effectiveness like St. John's wort for mild to moderate depression and Ginkgo biloba for mild cognitive impairment, the beneficial effect of most herbal supplements in modern medicine is at large due to limited, inconclusive, or mixed study results [5]. Rather, multiple pieces of evidence are emerging which demonstrate herbal supplements are becoming a public health concern for their potentially harmful effects [12]. There is also a regulatory loophole in controlling herbal products in the US as the Food and Drug Administration (FDA) does not require pre-marketing approval unlike conventional medications like prescription and over-the-counter drugs [13].

Since there exist huge knowledge and communication gaps regarding herbal supplements in the US conventional medical practice, the purpose of this comprehensive narrative review is to fill the gaps by summarizing scientific studies and relevant publications which address the clinical significance of herbal supplements particularly the health risks they pose. We also highlighted the multifaceted roles of physicians in mitigating the health risks of herbal supplements. Pertinent findings based on English text articles were retrieved from PubMed and Google Scholar databases. Websites operated by federal agencies were also explored to reflect on current US healthcare, research, and regulatory perspectives.

## Review

General overview of herbal supplements in the US

Definition

Herbal supplements (HS) are one type of dietary supplement (DS) containing one or more herbs that are available without a prescription. According to the Dietary Supplement Health and Education Act (DSHEA) of 1994, dietary supplements are broadly defined as products containing one or more dietary ingredients, including vitamins, minerals, herbs or other botanicals, amino acids, enzymes, tissues from organs or glands, or extracts of these which supplement the diet and taken by mouth (such as a tablet, capsule, powder, or liquid) [13,14]. There are different alternative terms used to describe herbal supplements such as botanicals, herbal products, herbal medicines, herbal remedies, and phytotherapy [14-16]. Herbal supplements in the form of tablets, capsules, powders, teas, extracts, and fresh or dried plants are sold and consumed to maintain or improve health [16].

Retail Channels

**Figure 1:**
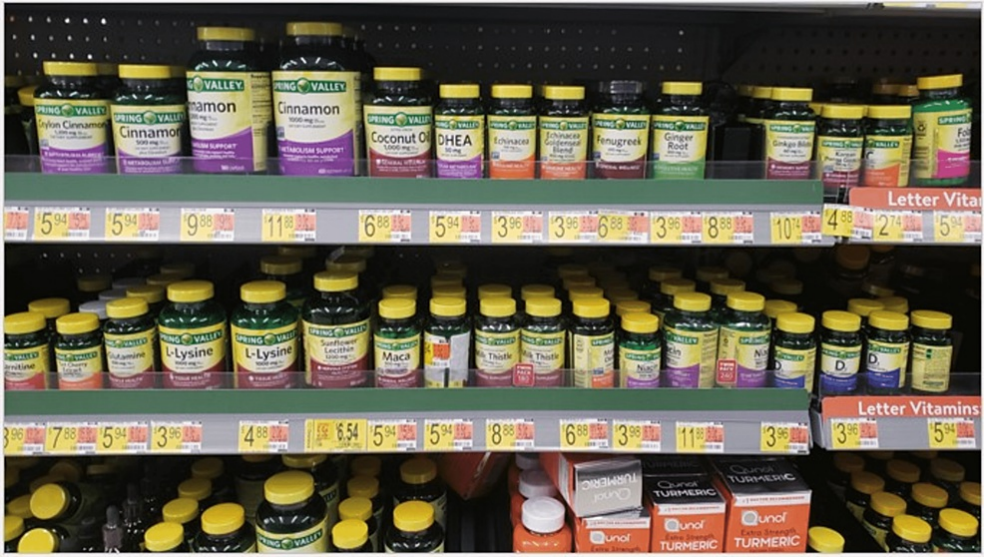
Example of mainstream retail channel for herbal supplements: Walmart aisle Figure Credit: Gashaw Hassen

**Figure 2:**
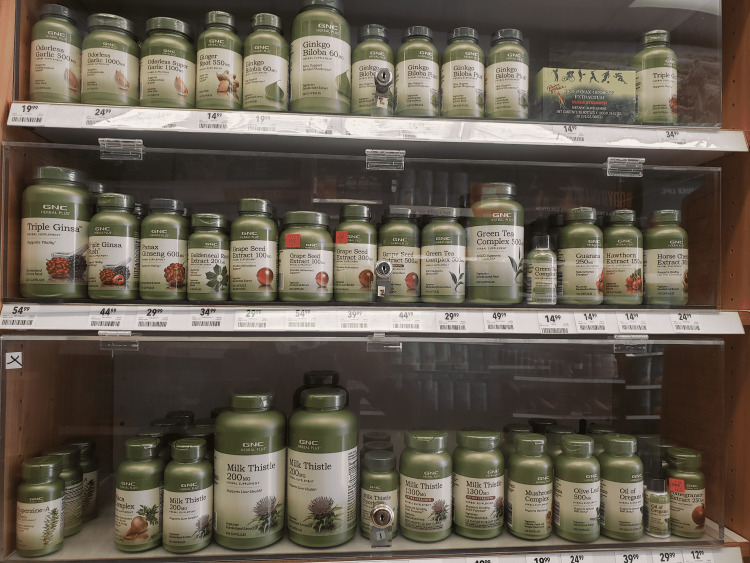
Example of natural retail channel for herbal supplements: GNC aisle GNC: General Nutrition Center Figure Credit: Gashaw Hassen

An estimated 20,000 or more herbal products are available in the US most lacking evidence of efficacy [17]. There are more than 50 herbs included in a series of fact sheets titled ‘Herbs at a Glance’ compiled by the US National Center for Complementary and Integrative Health (NCCIH) which provides basic herbal information for public awareness [18]. The two major US retail channels selling herbal products include mainstream retail channels such as grocery outlets, drug outlets, and convenience stores, and natural retail channels such as general nutrition centers (GNC), nutrition stores, and supplement retail outlets (Figure 1 & Figure 2). There are also direct-to-consumer sales of herbal products through E-commerce websites such as Amazone.com or direct selling media such as TV, radio, and print publications [11].

Predictors

In a recent systematic review on the potential factors that influence the use of CAM including herbal supplements amongst cancer patients and patients with other chronic illnesses, the most commonly reported reasons include benefits, safety, dissatisfaction with CM, influence by their social network, internal health locus of control, affordability, willingness to try/use, practitioners’ recommendation, easy access, holistic approach, and tradition. The same review also identified lack of information and trust, ineffectiveness, side effect concerns, and satisfaction with CM as some of the reasons for not using CAM [19].

Another study about the prevalence and predictors of herbal medicine use among adults in the United States based on the 2015 National Consumer Survey on the Medication Experience and Pharmacists' Role revealed that age older than 70, having a higher than high school education, using prescription medications or over-the-counter (OTC) medications, and using a mail-order pharmacy were associated with herbal use. Among herbal supplement users, there was concomitant use of prescription (38%) and OTC use (42%). Stroke (48.7%), cancer (43.1%), and arthritis (43.0%) were the most common conditions associated with herbal use. Among herbal product users, higher than school education, OTC medications use, mail-order pharmacy use, stroke, obesity, arthritis, and breathing problems were predictors of herbal use showing a higher adjusted odds ratio (AOR) [20].

Challenges

Multifaceted challenges/gaps pertinent to patients, providers, manufacturers, and regulators are contributing to the ill-health effects of herbal products. Patients not disclosing herbal use assuming "natural" is safe, providers not screening herbal use, weak premarketing regulation, impure herbs introduced to the markets, and misleading consumers through unethical marketing are among the major contributors to the negative impacts of herbs [13,21-24]. FDA is given a limited regulatory scope and doesn't enforce strict pre-marketing regulations for herbs which accelerated the booming of the herbal supplement industry since the passage of DSHEA in 1994 [25].

The National Institute of Health

In June 1993, the Office of Alternative Medicine (OAM) was formally established by the National Institute of Health (NIH) to study and evaluate CAM to disseminate the results to the public. In October 1998, the National Center for Complementary and Alternative Medicine (NCCAM) was established by Congress. In December 2014, Congress renamed NCCAM the National Center for Complementary and Integrative Health (NCCIH) [26]. Today, NCCIH, under NIH within the U.S. Department of Health and Human Services (HHS), is the lead Federal agency in scientific research on complementary and integrative health approaches [27].

The Office of Dietary Supplements (ODS), part of NIH, was established by Congress as part of the DSHEA of 1994 and is now the lead federal government entity that addresses the scientific exploration of DS with the mission of strengthening the knowledge and understanding of DS [28]. In response to the 1999 Congressional mandate, ODS in partnership with NCCIH initiated the Consortium for Advancing Research on Botanical and Other Natural Products (CARBON) Program to promote collaborative and transdisciplinary research on the safety, effectiveness, and mechanisms of action of botanical DS [29].

Manufacturers

According to Technavio, a leading global market researcher, the top US-based companies dominating the global herbal supplement industry include Gaia Herbs (North Carolina), Herb Pharm (Oregon), Nature’s Bounty (New York), Nutraceutical Corporation (Utah), Rainbow Light Nutritional Systems (California), Bio-Botanica Inc. (New York), and Arizona Natural Products (Arizona) [30]. Currently, more than 400 companies involved in the herbal industry as growers, processors, manufacturers, and marketers of herbs and herbal products are networked under a national trade association named the American Herbal Products Association (AHPA) which was first founded in 1982 [31].

Health Hazards

Herbs pose life-threatening pharmacological and toxicological health hazards [32,33]. These health effects arise from both biologically active intrinsic effects of the herbs themselves and the extrinsic toxic effects of the impurities invited by adulterants and contaminants [34]. The herb-drug interaction (HDI) is secondary to either the pharmacokinetically altered levels of the drug or its metabolites during absorption (A), digestion (D), metabolism (M), or excretion (E) (ADME) due to the presence of herbs or the pharmacodynamic effect of herbs mainly due to direct action on drug/molecular target rendering additive, synergistic or antagonistic changes in the pharmacological effects of the actual drug/drugs [35]. Besides, some herbs are genotoxic or carcinogenic (e.g., Aristolochia can cause A to T transversion and Upper tract urothelial cancer (UTUC)) [36-38].

The magnitude of herbal use in the US

According to the 2012 data from the National Health Interview Survey (NHIS) by the CDC, the national average for the use of nonvitamin, nonmineral dietary supplements was 17.9% which was the highest of any other complementary health approach. The Mountain (28.7%) and the South Atlantic (13.1%) US regions showed the highest and the lowest use prevalence respectively [39].

Based on the 2015 National Consumer Survey on a total of 26,157 eligible respondents, Rashrash et al. in their study on the prevalence of herbal medicine use among the US adults found that 35% (around one-third) reported current use of at least one herbal medicine and the average number of herbal supplements used was 2.6 [20].

The American Botanical Council (ABC), also known as the Herbal Medicine Institute, is a nonprofit research and education organization that reports on the herbal market in its quarterly journal (HerbalGram) [40]. According to ABC, the sale of herbal products exceeded US$5.3 billion in the United States in 2011 showing a 4% increase over 2010 [20,40].

In 2020, the herbal market hit a record high with double-digit growth of 17.3% of the previous year for the first time due to market demand fueled by COVID-19-driven herbal use for claimed immune health and stress relief. The annual sales of herbal supplements over the last two decades continued to increase and reached a record-breaking $11.261 billion spending in 2020, $1.659 billion more than in 2019 [11].

List of herbs consumed in the US

Fact sheets by NCCIH's Herbs at a Glance webpage, recently also launched as HerbList (App), published about the safety and effectiveness of 50+ popular herbal products marketed for health purposes to help consumers, patients, healthcare providers, and other users in quick access of research-based information (Table 1) [18]. Recently (in 2021), a natural product market research firm (SPINS) based in Chicago, Illinois, a natural products industry publication named Nutrition Business Journal (NBJ) based in Boulder, Colorado, and the ABC based in Austin, Texas jointly provided data on the US retail sales of herbal supplements. SPINS specifically reported on the 40 top-selling herbal supplements available in the US market which are published by HerbalGram, a quarterly journal of ABC (Table 1) [11]. Most of the herbs are promoted for claimed health benefits such as immune, cardiovascular, respiratory, digestive, prostate, and mental health (Table 2) [11,18,41-44]. The Colorado State University also published a fact sheet with a list of toxic herbs posing life-threatening side effects which are flagged by the FDA warning (Table 3) [15].

**Table 1:** Summary of herb lists retrieved from ABC and NIH ABC: American Botanical Council, NIH: National Institute of Health

Source	List of Herbs
ABC [11]	Elderberry, Horehound, Cranberry, Turmeric, Apple cider vinegar, Ginger, Echinacea, Garlic, Fenugreek, Wheatgrass / Barley grass, Saw palmetto, Ashwagandha, Green tea, Ivy leaf, Ginkgo, Cannabidiol (CBD), Black cohosh, Black cohosh, Red yeast rice, Aloe, St John’s wort, Flax seed / Flax oil, Milk thistle, Yohimbe, Goji berry, Valerian, Horny goat weed, Bioflavonoid complex, Beetroot, Cinnamon, Sennaf, Green coffee extract, Plant sterols, Ginseng, Chamomile, Garcinia, Fennel, Maca, Açaí, Rhodiola
NIH [18]	Acai, Aloe Vera, Asian Ginseng, Astragalus, Bilberry, Bitter Orange, Black Cohosh, Bromelain, Butterbur, Cat’s Claw, Chamomile, Chasteberry, Cinnamon, Cranberry, Dandelion, Echinacea, Elderberry, Ephedra, European Mistletoe, Evening Primrose Oil, Fenugreek, Feverfew, Flaxseed and Flaxseed Oil, Garcinia Cambogia, Garlic, Ginger, Ginkgo, Goldenseal, Grape Seed Extract, Green Tea, Hawthorn, Hoodia, Horse Chestnut, Kava, Lavender, Licorice Root, Milk Thistle, Mugwort, Noni, Passionflower, Peppermint Oil, Pomegranate, Red Clover, Rhodiola, Sage, Saw Palmetto, Soy, St. John’s Wort, Tea Tree Oil, Thunder God Vine, Turmeric, Valerian, Yohimbe

**Table 2:** Profile of the 10 top herbs sold by the US mainstream multi-outlet retail channels

S.N.	Herb Name	Common name	Latin name	Claimed health benefits	Side Effect / Safety
1	Elderberry	European elder, black elder, elderberry, elderflower, Sambucus	Sambucus nigra	colds, flu, COVID-19	nausea, vomiting, diarrhea
2	Horehound	white horehound	Marrubium vulgare, Lamiaceae	respiratory conditions, sore throat	nausea, oral dryness, sialorrhea, dizziness, anorexia
3	Cranberry	cranberry, American cranberry, bearberry	Vaccinium macrocarpon, Oxycoccus macrocarpos, Vaccinium oxycoccos	bladder, stomach, liver disorders, diabetes, wounds, urinary tract infections	stomach upset, diarrhea
4	Turmeric	turmeric, turmeric root, Indian saffron	Curcuma longa, Curcuma domestica; Curcuma aromatica	arthritis, digestive disorders, respiratory infections, allergies, liver disease, depression	Unsafe for pregnancy
5	Apple cider vinegar	ACV, cider vinegar, apple vinegar	Malus spp., Rosaceae	weight loss, blood sugar, and blood pressure regulation, digestive health, cholesterol reduction, immune support, skincare, “cleanse and detox”	hypokalaemia, hyperreninemia, osteoporosis, Oesophageal injury, skin irritation, chemical burns, enamel damage
6	Ginger	ginger	Zingiber officinale	nausea & vomiting associated with pregnancy	abdominal discomfort, heartburn, diarrhea, mouth & throat irritation
7	Echinacea	echinacea, purple coneflower, coneflower, American coneflower	Echinacea purpurea, Echinacea angustifolia, Echinacea pallida	common cold, topical use for wounds & skin problems	nausea, stomach pain, allergic reactions, skin rashes
8	Garlic	garlic	Allium sativum	Cardiovascular health (cholesterol & blood pressure control)	breath & body odor, heartburn, upset stomach, allergy, risk of bleeding, drug interaction
9	Fenugreek	fenugreek	Trigonella foenum-graecum	diabetes, menstrual cramps, stimulate milk production during breastfeeding	diarrhea, nausea, dizziness, headaches, hypoglycemia, allergic reactions, hepatotoxicity, birth defects
10	Wheatgrass	Wheatgrass juice	Triticum aestivum	ulcerative colitis	nausea, constipation

**Table 3:** Toxic herbs causing life-threatening side effects which warrant closer attention

S.N.	Herb Name	Side Effects
1	Aristolochic Acid	nephrotoxic, carcinogenic
2	Chaparral	irreversible liver damage
3	Comfrey	hepatotoxic, carcinogenic, teratogenic
4	Ephedra/ma huang (ephedra sinica)	hypertension, myocardial infarction (MI), seizure, stroke, psychosis
5	Germander	hepatotoxic, death
6	Kava	hepatotoxic
7	Lobelia (Indian tobacco)	breathing problems, tachycardia, hypotension, coma, death
8	Magnolia-Stephania	kidney disease, permanent kidney failure
9	Willow bark	Reye’s syndrome in children, allergic reaction in adults
10	Wormwood	seizures, numbness of extremities, delirium, kidney failure
11	Yohimbe	BP irregularity, arrhythmia, kidney & neurologic disorders, death

Regulation and quality control of herbs in the US

DSHEA & FDA

The DSHEA of 1994 with Public Law No. 103-417 1994 first defined dietary supplements to include herbs [45]. The two federal agencies are mainly involved in the regulatory aspects of DS; the FDA regulates the quality, safety, and labeling, and the Federal Trade Commission (FTC) monitors the advertisements and marketing [25].

According to the DSHEA, FDA received jurisdiction to regulate herbal products but its scope of regulation is limited mostly to post-marketing surveillance carrying the burden of proof in demonstrating that these products pose significant health risks before removal from the market [25,46]. Unlike conventional drugs, manufacturers are not expected to prove the safety of herbs before marketing unless they are introducing new ingredients identified after 1994. Enforcing the notification of new ingredients by the FDA is also very challenging. Despite a dramatic jump in the total sale of DS from 4000 in 1994 to 90,000 in 2014, FDA was notified of new ingredients only in 170 DS cases in two decades (1994-2012) [25].

Without undergoing evaluation as conventional drugs by FDA, manufacturers of herbal supplements are prohibited from making health claims about their products’ ability to diagnose, mitigate, treat, cure, or prevent a specific disease or class of diseases [13,45,47]. By law, manufacturers and distributors of herbal supplements and ingredients are prohibited from marketing adulterated or misbranded products [48]. The FDA requires manufacturers to include the following information on labels: name of the product or supplement, name and the address of the manufacturer or distributor, complete list of ingredients, and amount of product or supplement in the container or package. The statement “Not evaluated by the FDA. Not intended to diagnose, treat, cure, or prevent any disease” should also be incorporated on the labels [47,49]. Permissible health claims characterizing how the dietary supplement (herb) maintains the normal structure or function in humans can be incorporated into the labels [25,47]. Supplement companies must uphold that claims on the labels of their products must be truthful and not misleading [50]. FDA also requires manufacturers to inform any adverse events reported directly to them [47-50].

In 2007, the FDA established non-binding current good manufacturing practice (cGMP) guidelines to ensure consistency in product quality in terms of identity, purity, strength, and composition of dietary supplements. According to cGMP, manufacturers are required to test the quality of products, confirm products are free from some contaminants, verify the accuracy of product labeling, adhere to the minimum manufacturing and packing standards, closely monitor reports of adverse events (AE), and avail all records for FDA inspection [25,50-52]. However, cGMP guidelines still lack to ensure the safety of the supplements as manufacturers are not uniformly adopting them [52,53].

The FDA can remove herbal supplements from the market if they are found to be unsafe, adulterated, or misbranded with false or misleading labels [49,54]. FDA states that it will evaluate the marketplace and take action on unsafe herbal supplements to protect the public if consumers, healthcare professionals, manufacturers, packers, distributors, and researchers submit adverse reactions through the Safety Reporting Portal (SRP) [55,56]. Consumers can also contact the local FDA Consumer Complaint Coordinator assigned for the state using the provided numbers to report serious reactions or illnesses which arise from using herbal supplements [57]. In addition to the Center for Food Safety and Applied Nutrition (CFSAN) dedicated to the FDA mission, and a flagship alert system, MedWatch, the FDA issues warning letters, recalls, seizures and injunctions, press announcements, public notifications, and safety alerts for health fraud scams involving products with unproven claims [58]. FDA randomly and periodically inspects manufacturing facilities or supplements [59].

Quality Assurance

ConsumerLab.com, NSF International, and U.S. Pharmacopeia are some of the independent organizations in the US which offer quality testing and allow manufacturers to display a seal of quality assurance if their herbal products pass the tests [59]. Electron microscopy, thin layer chromatography (TLC), high-performance liquid chromatography (HPLC), liquid chromatography-mass spectrometry, and DNA barcoding are newer techniques applied in biological testing used to evaluate the quality of herbal products [60-62]. Since imported herbal supplements pose questionable regulations, it is advised to purchase quality-tested products that are made in the US and available in established outlets [63].

Ephedra: The First Banned Supplement

Ephedra (ma huang) which contains ephedrine alkaloids became the first supplement banned by the FDA in February 2004 for posing unreasonable risks of illness or injury to the public [64]. According to the 68-page final rule, FDA stated the action of prohibiting the sale of ephedra was based on the “well-known pharmacology of ephedrine alkaloids, the peer-reviewed scientific literature on the effects of ephedrine alkaloids, and the adverse events reported to have occurred in individuals following consumption of dietary supplements containing ephedrine alkaloids” [65]. Marketed as a weight-loss and energy-enhancement agent in the US during the 1990s and early 2000s, Ephedra caused fatal cardiovascular and neurologic effects including myocardial infarction, stroke, and seizure through its direct adrenergic stimulation and indirect endogenous catecholamine release. A 13-year post-ban follow-up showed a dramatic decline in ephedra-related total deaths and poisoning reports [66].

The Federal Trade Commission

The Federal Trade Commission (FTC) protects consumers and competition from deceptive, and unfair business practices through law enforcement, advocacy, and education [67]. Each year, the FTC enforces laws against hundreds of individuals and businesses involved in fraud, scams, identity theft, false advertising, privacy invasion, anti-competitive behavior, and more [68]. Along with FDA, FTC also makes consumers aware of tainted products, recalls, alerts, and advisories [69]. It is common to see problematic advertising of herbal products promoted with false claims which mislead consumers and pose safety issues. Recently, FTC sent warning letters to more than 120 marketers to stop making unsubstantiated claims that their products and therapies can treat or prevent COVID-19 [70].

Following is the summary of herbal regulation and quality control in the US (Table 4) [71].

**Table 4:** Regulatory and quality landscape of the US herbal supplements DSHEA: Dietary Supplement Health and Education Act, DS: Dietary Supplement, FDA: Food and Drug Administration, cGMP: current good manufacturing practice, PO: Per Os (by mouth)

Regulatory authorization	Premarketing safety data	cGMP	Label requirements	Permissible health claims
DSHEA of 1994 classifies herbs as DS (intended for PO) and restricts FDA from strict premarketing regulation	Only for ingredients introduced after 1994	➤Manufacturing ➤Packaging ➤Labeling	➤ Name of each ingredient ➤ Quantity of each ingredient ➤ Manufacturer’s contact information ➤The statement: “Not evaluated by the FDA. Not intended to diagnose, treat, cure, or prevent any disease”	➤Not required to be preapproved ➤Characterize how DS maintains normal structure or function in humans

Sources of adulterants and contaminants in herbal supplements

There is a growing concern among the public and medical community about health risks of contaminants such as heavy metals, microbial pathogens, pesticide residues, and misidentified plants as well as adulterants such as prescription medications, toxic plant extracts, and inappropriate additives introduced during agricultural processing, product preparation and packaging (Table 5) [72].

**Table 5:** Summary of impurities related to herbal products

Extrinsic Source of Toxicity	How?	Examples
Heavy Metals	agricultural soil, irrigation water, air pollution, fertilizers, herbicides & pesticides	Aluminum, Arsenic, Cadmium, Copper, Lead, Mercury, Thallium, Tin, Zinc
Pharmaceuticals	adulterants, hidden ingredients, additives, fillers	Prescription and over-the-counter drugs
Microbes	field and post-harvest contaminations	Toxic bacteria and fungi

A study by the California Department of Health Services, Food and Drug Branch on 260 Asian patent medicines collected from California retail herbal stores revealed 17 (7%) contained undeclared pharmaceuticals such as ephedrine, chlorpheniramine, methyltestosterone, and phenacetin purposefully and illegally added to achieve the desired effect. Significant amounts of heavy metals were also identified including lead, arsenic, and mercury in 24 (9%), 36 (14%), and 35 (14%) products respectively. Moreover, 23 (9%) had more than one adulterants [17,73]. The toxicities introduced by heavy metals, prescription drugs, or unapproved ingredients pose serious health hazards to unsuspecting consumers [74-77].

To protect consumers, US retailers such as CVS launched a third-party independent evaluation for contaminants such as heavy metals, yeast, mold, pesticides, biological pathogens, and industrial compounds like polychlorinated biphenyls on DS supplied by makers and distributors before they are available in their stores or online [78].

Significance and burden of herbal supplements in clinical practice

Undesirable Health Outcomes

According to the national estimates of using natural product supplements for wellness purposes among U.S. adults in 2012, general wellness or disease prevention (83.3%), improve immune function (42.0%), improve energy (31.0%), focuses on the whole person (mind, body, and spirit) (26.5%) and improve memory or concentration (22.2%) were among the top reasons mentioned [79]. Though perceived as safe due to their natural source, herbal supplements constitute different side effects and adverse health outcomes originating from biologically active herbal constituents, contaminants, and herb-drug interactions (HDI) [17].

A nationally representative surveillance data of 3667 cases obtained from 63 US emergency departments analyzed for 2004-2013 visits estimated that 23,005 (95% CI: 18,611-27,398) annual visits in the US were attributed to adverse events related to DS resulting in an estimated 2154 hospitalizations (95% CI: 1342-2967) annually. Herbal or complementary nutritional products used for weight loss (25.5%; 95% CI: 23.1-27.9) and increased energy (10.0%; 95% CI: 8.0-11.9) were commonly implicated causing 71.8% (95% CI: 67.6-76.1) of adverse events including palpitations, chest pain, or tachycardia [80].

A study on 105 morbid case series of Chinese-herb nephropathy seen as an outbreak in Belgium among users of weight-reducing pills inadvertently manufactured with nephrotoxic and carcinogenic Aristolochia fangchi showed end-stage renal failure (43 cases of which 39 had prophylactic kidney removal) and urothelial carcinoma due to DNA adducts (18 cases) [17,81].

Using herbal remedies as galactagogues and as therapies for postpartum conditions such as constipation, postpartum depression, and upper respiratory tract infection by breastfeeding women may constitute health risks to the infant due to herbal chemical constituents, contaminants, and heavy metals which enter human milk [82]. It is also common to see the use of herbal supplements during pregnancy to improve the wellbeing of the mother and/or baby, reduce nausea and vomiting episodes, treat infections, relieve gastrointestinal problems, facilitate labor or ease labor pains. Pregnant women with pre-existing conditions like epilepsy and asthma are at increased risk of potential herb-drug interactions. Besides, theoretical concerns exist about some herbs' teratogenic or embryotoxic effects [83,84].

Drug-induced liver injury (DILI) is the most difficult form of liver disease diagnosed by exclusion. The challenges of DILI are mainly due to its occurrence without warning, the absence of specific distinguishing features or markers, and its mimicry of multiple liver diseases [85]. An Icelandic study revealed that 16% of DILI was attributed to the use of herbals and dietary supplements (HDS) whereas the US DILI Network (DILIN) study on the hepatotoxicity caused by conventional medications and HDS showed an increase in liver injury caused by HDS from 7% to 20% during 2004-2013 study period resulting in hospitalization, liver transplantation and even death [85,86].

A US survey of 500 ambulatory surgical patients revealed that 42.7% of them took alternative medicines in the two weeks period before their surgical procedure which had significant implications for both anesthesia and surgical care. Further analysis of the survey showed these CAMs inhibit coagulation (19.8%), affect blood pressure (14.4%), and result in both cardiac (7.4%) and sedative (8%) effects [87]. Harmful interactions of herbal supplements with conventional medicines through the cytochrome P450 pathway systems may also have potentially deleterious effects during the perioperative period and wound healing [88].

The effects of herbal supplements on laboratory test results are many folds. Abnormal liver function tests (LFT), renal function tests (RFT), and thyroid function tests (TFT) are related to significant organ damage. Kava-Kava, chaparral, germander, and mistletoe for example can cause abnormal LFT secondary to liver damage. An abnormal level of therapeutic drug reflected during routine monitoring may reveal unexpected HDI. Lead poisoning can occur after consuming DS contaminated with heavy metals. A patient may show a falsely abnormal digoxin level when digoxin and certain Chinese medicine such as Chan Su or Lu-Shen-wan are taken together due to direct interference of a component of Chinese medicine with the antibody used in an immunoassay [89].

Herbal Supplements to Avoid During Surgery, Pregnancy, and Breastfeeding

The following table (Table 6) summarizes the list of herbal supplements to avoid during surgery, pregnancy, and breastfeeding for the negative and deleterious effects they pose on anesthesia, surgery, pregnancy outcome, nursing, and postnatal life [84,88,90-92].

**Table 6:** List of published herbal supplements to be avoided during the perioperative period, pregnancy and breastfeeding SJW: St. John's Wort

Avoid When?	Herbal supplements to avoid	Reasons for avoiding the herbal supplements
Within two weeks of surgery [88,90]	Gingko biloba, Garlic, Ginseng, Dong Quai, Feverfew	Bleeding effects
	Ephedra, Garlic	Cardiovascular effects: Ephedra (tachycardia, hypertension, and palpitations), Garlic (hypotension)
	Kava, Valerian root, SJW	Anesthetic effects
	Echinacea, Goldenseal, Licorice	Cytochrome P450 inhibitors (interacts with coumadin, cyclosporine, midazolam, oral contraceptives, testosterone, lidocaine, and digitalis)
	SJW	Cytochrome P450 inducers
	Kava, Valerian root	Effects on sedatives (benzodiazepine and barbiturates)
	SJW and Dong Quai	Photosensitivity
	Ginseng	Hypoglycemia
During Pregnancy [91,92]	Ammi visnaga, Blue cohosh, Cat's Claw, Fenugreek, Feverfew, Pennyroyal, Sage, Thyme	Uterotonic effect causing preterm birth or abortion
	Andrographis, Boldo, Catnip, Essential oils, Feverfew, Juniper, Licorice, Nettle, Red clover, Rosemary, Shepherd's purse, and Yarrow	Caution is needed during pregnancy due to safety concerns
	Dong Quai	Negatively affect the fetus
	Soy, Isoflavones, Red clover, Flaxseed, Lignans, and Hops	Estrogen-like properties with possible effects on the fetus
	Green tea	Theoretical concern for risk of birth defects due to effect on folate levels
	Traditional Chinese herbal combinations and Ayurvedic herbal combinations.	Associated toxic heavy metals, poisonous herbs, or unlabelled prescription drugs may damage the intrauterine growth or affect the lactating baby through milk
During Breast Feeding [84,92]	Chasteberry	Milk-inhibiting potential
	Aloe	Laxative effect due to anthraquinones glycosides excreted with breast milk
	Black Cohosh	Gastrointestinal irritation in baby
	Butterbur	Hepatotoxic effect due to pyrrolizidine alkaloids excreted with breast milk.
	Ephedra	Stimulants excreted in breast milk
	Goldenseal	May raise the infant bilirubin levels
	Kava Kava	CNS depressant due to pyrones in the breast milk
	Licorice root	Potential toxicity to the infant
	Senna leaf	Genotoxic anthraquinones excreted in breast milk
	Wormwood	Potential neurotoxins excreted in breast milk

Herbal Adverse Reactions

The traditional classification of adverse drug reactions (ADRs) intended for conventional/synthetic medicine can be applied to further describe the adverse effects of herbal supplements (Table 7) [72, 93-95]. Adverse drug event (ADE) is sometimes used as an umbrella term that includes ADRs and medication errors (MEs) [96]. According to the World Health Organization (WHO) definition, ADR is implied to any noxious, unintended, and unwanted response to a drug used for treatment, prophylaxis, or diagnosis at normal doses [96]. A policy publication by Walji et al. also stated the 1995 definition of ADRs by WHO as ‘unintended consequences suspected to be related to the use of medicinal products, including herbal medicines’ [97].

**Table 7:** Traditional classification of herbal adverse reactions

Type of Reaction	A (acute / augmented)	B (bizarre / idiosyncratic)	C (chronic / cumulative)	D (delayed)
Characteristics	dose-dependent and predictable, common, preventable by dose reduction	idiosyncratic and allergic, unpredictable, no correlation between dose level and risk of toxicity, dose/time-independent, affects small population (rare), often serious and potentially fatal, environmental and genetic dependent	develop during long-term therapy, well-described, and anticipated reaction	delayed effects, such as mutagenicity, carcinogenicity, and teratogenicity
Example	Yohimbine (hypertension and anxiety), Kava (hepatotoxicity)	Yohimbine (bronchospasm and mucus hypersecretion), Echinacea (acute asthma, hives, and anaphylaxis)	herbal anthranoid laxative and, licorice (hypokalemic paralysis)	Aristolochia (Urothelial carcinogenic), Pyrrolizidine alkaloids containing herbs such as Symphytum officinale (Hepatocarcinogenic, teratogenic liver failure), Acorus calamus, Aristolochia species, Blighia sapida, Gonium macula tum, Croton tiglium, Genista tinctoria, Sassafras albidum (carcinogenic in animal studies),

Over 30% of all ADRs are caused by drug-drug interactions (DDIs), causing serious morbidity each year which warrants early detection [98]. Similarly, due to the upsurge of herbal supplements which are used alone or co-administered with conventional drugs, adverse reactions and drug interactions attributed to herbal supplements are becoming a significant safety concern [35,99].

Due to poor regulation of herbal products, impurities like allergens, pollen, and spores and batch-to-batch variability could contribute to some of the reported herb-related adverse effects and drug interactions [100]. Information about potential herb-related adverse reactions is very limited [101]. Estimates of adverse events related to dietary supplements (including herbs) suggest that only 1% of cases are reported to the FDA [102]. A manual review of case reports from PubMed identified approximately 21, seven, and seven herbs as being risks for liver, kidney, and heart toxicity, respectively [101]. Currently, advancement in modern technology is continually improving how ADRs are predicted, prevented, detected, and managed [95].

Herb-Drug Interaction (HDI)

Concurrent consumption of herbal supplements and conventional drugs results in HDI which has important clinical significance in medical practice [103]. Drugs with a narrow therapeutic index (such as warfarin and digoxin) and drugs prescribed for the long-term treatment of chronic or life-threatening illnesses raise serious safety concerns when coadministered with herbal supplements [35]. As evident in multiple case reports, case series, and pharmacokinetic trials, herbal supplements and prescribed drugs may interact in the intestine, liver, kidneys, and targets of action of which up or down-regulation of cytochrome P450s and/or P-glycoprotein are most prominent [104].

Pharmacokinetic HDI mechanisms affecting drug absorption, induction and inhibition of metabolic enzymes and transport proteins, and changes in renal excretion of drugs alter the level of drugs and their metabolites which can be countered by safe adjustment of the drug. Moreover, less common pharmacodynamic HDI mechanisms due to intrinsic pharmacologic properties of herbal supplements acting on drug sites/receptors can result in synergistic, additive, and/or antagonistic effects of the concomitant drug without alteration of its level and are also unlikely countered by a change in drug dosage (Table 8) [35,103,105]. It is worth noting that patient factors such as age, gender, and pathological status; drug factors such as dose, dosing regimen, and route of administration; and herb factors such as herb pretreatment, herb-herb interactions, and chemical and physical modification of herb ingredients are among the potential influencers in the pharmacokinetics of herbal supplements [106].

**Table 8:** Summary of HDI, mechanisms, and outcomes GI: Gastrointestinal, H-D: Herb-Drug, AKA: also known as, P-gp: P-Glyco protein, MDR1: multi-drug resistance protein 1, MRP2: Multi-drug resistance-associated protein-2, BCRP: Breast cancer resistance protein, CYP: cytochrome P450, UGTs: Uridine 5'-diphospho-glucuronosyltransferase

Herb-Drug Interaction	Level of Interaction	Mechanism	Herbal Effect	Outcome
Pharmacokinetic (ADME)	Absorption (A)	efflux transporters (P-gp AKA MDR1, MRP2, BCRP)	inhibition/induction of efflux transporters located at the canalicular membrane of epithelium or endothelium	change in the blood concentration of the drug
		GI motility	increase GI motility shortening the drug transit time and lowering absorption	
		insoluble H-D complex formation in GI tract	decrease drug bioavailability to a sub-therapeutic state	
	Distribution (D)	drug binding protein	displace protein-bound drugs increasing bioactive concentration	
	Metabolism (M)	CYP enzyme family (phase I) and non-CYP enzyme systems such as UGTs (phase II)	induction/inhibition of the metabolic enzymes in a competitive or non-competitive manner	
	Elimination (E)	renal (major) and biliary (minor) elimination of drugs	increase renal excretion of drugs and metabolites	
Pharmacodynamic	Drug Target	simultaneous effects on the same drug sites/receptors	changes in the physiological effect and mechanism of action of the drug	additive, synergistic or antagonistic changes in the pharmacological effects of the drug

HDI Screening Tools/Checkers

Despite an ever-increasing use of dietary supplements including herbal products, only 12-14% of users in Canada and the United States are reporting adverse effects [107,108]. With the advent of the internet, there are several online screening tools designed to check HDI to minimize or intervene in adverse events. These tools are available as web pages with unique Uniform Resource Locators (URLs) and or as mobile applications either for free or subscription-based access which can be consulted by herbal consumers, clinicians and researchers in order to determine HDI (Table 9) [104,107,108].

**Table 9:** Free and subscription-based online HDI screening tools Web: Webpage, App: Mobile Application, HDI: Herb-Drug Interaction, URL: Uniform Resource Locator

HDI Screening	Interaction Checker	URL Link for Online Access	Platform Availability
Free tools	Drugs.com	https://www.drugs.com/drug_interactions.html	Web & App
	Medscape	https://reference.medscape.com/drug-interactionchecker	Web & App
	WebMD	https://www.webmd.com/interaction-checker/default.htm	Web & App
	RxList	https://www.rxlist.com/drug-interaction-checker.htm	Web
	Merck Manual	https://www.merckmanuals.com/home/druginformation/drug-interactions	Web & App
Subscription-based tools	Micromedex	https://www.micromedexsolutions.com/home/dispatch/ssl/true	Web & App
	Lexicomp	https://www.wolterskluwer.com/en/solutions/lexicomp/lexicomp	Web & App
	Facts and Comparisons	https://www.wolterskluwer.com/en/solutions/lexicomp/facts-and-comparisons	Web
	PEPID	https://www.pepid.com/	Web & App
	Natural Medicines	https://naturalmedicines.therapeuticresearch.com/	Web

Patient Safety and Potential HDI of Common Herbal Supplements

The following table (Table 10) summarizes the clinically most relevant herbal supplements consumed among the US population based on the data retrieved from NCCIH Clinical Digest for health professionals. It highlights the health claims the herbal supplements are promoted in the market, the side effects they pose on patients, and potential HDI obtained from multiple scientific works of literature [72,100,105,109-122].

**Table 10:** Summary of adverse effects and herb-drug interactions of the most common herbs SJW: St. John’s Wort, INR: International Normalised Ratio, CYP: Cytochrome P-450 enzymes, P-gp: P-glycoprotein, BP: Blood Pressure, HR: Heart Rate, PAD: Peripheral Artery Disease, CCB: Calcium Channel Blockers, HA: Headache, N: nausea, V: vomiting, ADHD: attention-deficit hyperactivity disorder, OCD: obsessive-compulsive disorder, GI: Gastrointestinal, ICB: intracerebral bleeding, BF: Breast Feeding, OC: Oral Contraceptives

Herb Name (Latin/Scientific)	Promoted for	Adverse Effect	Herb-Drug Interaction	
			Interacting Drug	Effect of Interaction
Black Cohosh (Actaea racemosa, Cimicifuga racemosa)	hot flashes, other menopausal symptoms	stomach upset, cramping, HA, rash, feeling of heaviness, vaginal spotting or bleeding, weight gain	statins	reduced effectiveness
Garlic (Allium sativum)	high blood cholesterol, high BP	odor (breath & body), heartburn, stomach upset, risk of bleeding	Chlorpropamide	hypoglycemia
			Paracetamol	changes in pharmacokinetic variables
			Warfarin	increased INR
			Saquinavir	decreased concentration
Ginkgo (Ginkgo biloba)	anxiety, allergies, dementia, eye problems, PAD, tinnitus	HA, stomach upset, dizziness, N, V, palpitations, constipation, allergic skin reactions, bleeding risk, liver & thyroid cancer (animal study), early labor or extra bleeding during delivery (in pregnancy), ICB	Aspirin	spontaneous hyphaema
			Thiazide diuretic	increased BP
			Trazodone	coma
			Warfarin (Coumadin)	increased bleeding risk
					Ginseng, Asian (Panax ginseng)	to increase resistance to stress (adaptogen), for general well-being (general tonic), to improve physical stamina, concentration & memory; to stimulate immune function; to slow the aging process; to relieve respiratory & cardiovascular disorders, depression, anxiety, menopausal hot flashes, premature ejaculation (topical)	insomnia, menstrual problems, breast pain, increased HR, high or low BP, HA, loss of appetite, digestive problems, altered blood sugar, birth defects (animal study), questionable safety for infants, children, pregnancy or BF, platelet inhibition, lowering blood glucose	Drugs metabolized by CYP3A4 (Ginseng induces CYP3A4 enzymes)	decrease the effectiveness of drugs such as CCB, some chemotherapeutic & HIV agents, certain antihypertensive & statin medications, some antidepressants
Warfarin	decreased INR						
Goldenseal (Hydrastis canadensis)	colds and other respiratory tract infections, allergic rhinitis, ulcers, digestive upsets such as diarrhea and constipation, mouthwash, eyewash	unsafe for pregnancy or breastfeeding, neonatal jaundice	Metformin	drop in metformin level
			Drugs metabolised by CYP2D6 and CYP3A4 (Goldenseal inhibits both CYP2D6 & CYP3A4)	increase in the level of many pharmaceutical agents currently in use
SJW (Hypericum perforatum)	depression, menopausal symptoms, ADHD, somatic symptom disorder, OCD, topical use for skin conditions (wounds & bruises) & muscle pain	GI disturbances, allergic reactions, fatigue, dizziness, confusion, dry mouth, photosensitivity/phototoxicity (skin rash, nephropathy), insomnia, anxiety, headache, or sexual dysfunction, unsafe for pregnancy or BF (infantile colic, drowsiness & fussiness)	Drugs with pharmacokinetics involving CYP3A4 and P-gp (SJW is a potent inducer of CYP and intestinal P-g)	reduction in cyclosporine, indinavir, nevirapine, OC, warfarin (reduce INR), digoxin, ivabradine, benzodiazepines, tacrolimus, irinotecan, imatinib theophylline, venlafaxine, statins
			Certain antidepressants	serotonin syndrome

Case Reports Showcasing the Health Hazards of Herbs

Since randomized controlled trials (RCTs) could not reliably identify an ever-increasing incidence of rare adverse events emerging with the use of herbal supplements, case reports, case series, and post-marketing surveillance studies fill the evidence gap. Case reports coupled with pharmacokinetic trials provide the highest level of evidence. The pitfalls of case reports include the inability to establish causal relationships and the limited number of cases showing severe clinical reactions [104].

The table below (Table 11) is a summary of published case reports with key information retrieved to demonstrate the circumstances behind the adverse events attributed to herbal supplements [123-135].

**Table 11:** Previously published case reports showing herbal adverse effects YO: Years Old, Hx: History, Bx: Biopsy, DM: Diabetes Mellitus, HTN: Hypertension, PHTN: Pulmonary Hypertension, CVA: Cerebrovascular Accident, AFib: Atrial Fibrillation, INR: International Normalized Ratio, HIV: Human Immunodeficiency Virus, OA: Osteoarthritis, SJW: St. John's Wort, M: Male, F: Female, ART: Antiretroviral Therapy, ZDV: Zidovudine, 3TC: Lamivudine, NVP: Nevirapine, CYP: Cytochrome P450, P-gp: P-glycoprotein 1, Pb: Lead, DILI: Drug-Induced Liver Injury, HILI: Herb-Induced Liver Injury, Lab: Laboratory, CT: Computed Tomography, SVT: Supraventricular Tachycardia, ECG: Electrocardiogram, HR: Heart Rate, ED: Emergency Department

Author Name and Year of Publication	Case Description	Herb name, (Purpose of use)	Form / Dose / Duration of Herbal use causing the adverse effect	Interacted Drug	Mechanism of Adverse Effect
Alscher and Klotz (2003) [123]	57 YO, M, a kidney transplant patient, developed a sudden drop in immunosuppressant (Cyclosporin).	SJW, (depression)	regular drinking as a tea form	Cyclosporine	CYP3A4 and P-gp induction
Leung et al., (2008) [124]	80 YO, Chinese woman, with Hx of DM, HTN, CVA & AFib experienced two episodes of an elevated INR.	Goji berry / Lycium barbarum L (longevity)	After 1-2 days of use as tea	Warfarin	CYP2C9 inhibition & /or additive anticoagulation
Hamann et al., (2011) [125]	46 YO, African American woman, with a Hx of stage 1 sarcoidosis, uterine fibroids, anemia, cardiomyopathy, & depression developed increased INR.	Cranberry juice, (constipation)	2 quarts daily for 3-4 days	Warfarin	Unknown
Mateo-Carrasco et al., (2012) [126]	56 YO, a White Caucasian man, with a Hx of HIV, developed generalized pruritus, scratching lesions, increased transaminase, visible jaundiced skin, & mucous membranes (Raltegravir-induced liver injury).	Ginseng, (sexual disability)	After 39 days of use as oral lozenges	Raltegravir	CYP3A4 inhibition
Sobieraj and Freyer, (2010) [127]	58 YO, Mexican man, with a Hx of type 2 DM, OA, hyperlipidemia, HTN, & degenerative disc disease of the spine reported four hypoglycemic events.	Prickly Pear Cactus (PPC), (glucose control)	daily for two months as crude PPC pads	Glipizide, Metformin	Additive effect with antihyperglycemic agents
Cordova et al., (2017) [128]	49 YO, F, HIV+ with excellent adherence and sustained (10 yrs) virologic suppression developed two consecutive detectable viral loads (96 & 57 copies/mL).	“horsetail” / Equisetum arvense, (analgesic & anti-kidney stone)	two months before the first detectable viral load	ZDV, 3TC & NVP	Limited data, probably negative interaction with ART
Sorbera et al., (2017) [129]	70 YO, Haitian man, with AFib & PHTN, developed elevated INR.	Mauby, (Caribbean folk remedy)	recent use as a bitter liquid	Warfarin	No clear information
Prescott and Smereck, (2019) [130]	49 YO, M, presented to ED with features of Hypertensive Urgency including palpitations & severely elevated BP uncontrolled with multiple doses of oral clonidine & IV labetalol (refractory HTN).	Yohimbine, (enhance male sexual performance & improve energy level)	two capsules the morning of presentation to the ED	Not mentioned	Selective ⍺2- adrenoceptor antagonist
Koenig et al., (2021) [131]	53 YO, F, presented to the ED with features of HILI including marked hyperbilirubinemia with liver enzyme elevations (cholestatic jaundice); imaging revealed hepatomegaly & steatosis without biliary dilatation; Bx results consistent with DILI.	Scute root- Bupleurum- -Turmeric root Mix, ("Liver Detoxifier & Regenerator") & Valerian, ("Restful Sleep")	daily use for 3 to 4 weeks	Not mentioned	Hepatotoxicity
Mowafy et al., (2021) [132]	56 YO, M, Hx of DM & bilateral knee OA presented to ED with features of acute pancreatitis including elevated lipase (Lab) & acute peripancreatic inflammatory changes (CT).	Fenugreek, (decrease blood sugar)	Extended regular use	Metformin (resorted)	Inflammatory effect on the pancreas
Fisher et al., (2021) [133]	33 YO, F, with a reported Hx of depression developed SVT (Sweating, insomnia, frequent brief Palpitations, tachycardia of 150-160 & ECG revealing SVT with HR of 148 bpm).	SJW, (depression)	300 mg tab daily for three weeks	Not mentioned	Neurotransmitter modulation
Kupiec and Raj, (2005) [134]	55 YO, M, suffered a fatal breakthrough seizure while swimming, no evidence of noncompliance with his anticonvulsant medications. An autopsy report revealed subtherapeutic serum levels of anticonvulsants (valproic acid & phenytoin).	Ginkgo biloba, (CVA)	undisclosed use around one year before his death	Valproic acid (Depakote) & Phenytoin (Dilantin)	Potent neurotoxin from Ginkgo nuts & Induction of CYP enzyme system
Gardiner et al., (2013) [135]	A systematic review of 128 pediatric case reports (40 from the US), to evaluate herbal adverse events, in <18 YO cases, published during 44 yrs (1965-2008). Events include: Neurologic (45), Gastrointestinal (18), Liver toxicity & jaundice (14), Cardiovascular/hematological (13), Dermatological (12), Respiratory (9), Endocrine/reproductive/renal (8), Cyanosis (6), Neonatal withdrawal (2), Anaphylactic shock (1)	Various herbal products, (prenatal (8) vs. postnatal (118) exposure, unintentional exposure (47) vs intentional use (59) for pediatric health	Pregnancy use (prenatal exposure), ingestion, and skin exposure	Not mentioned	Organ damage, hormonal imbalance, Pb toxicity

The roles of physicians in minimizing health risks of herbal supplements

With an ever-increasing use of herbal supplements along with prescription and over-the-counter medications, a prudent clinical practice should embrace identifying susceptible groups and safer treatment plans which should monitor and mitigate any possible adverse effects [95,136]. Physicians should be cognizant of purported adverse effects and deleterious drug interactions associated with herbal supplements and should ask all patients about the use of these products as part of their medication history [100]. An anonymous survey to determine the physicians’ knowledge regarding the toxic effects and drug interactions of herbal remedies revealed that they are provided with little training in herbal toxicities and drug interactions and hence are poorly familiar with these topics [137].

The 2012 National Health Interview Survey (NHIS) based on 34,525 adult respondents revealed that around 30% used CAM at least once in the past year and 66% had Primary Care Physician (PCP). Then, further analysis based on 7493 respondents who both use CAM and had PCP showed around 42% did not disclose their most used CAM modality including herbal supplements (in 25%) attributing the non-disclosure mainly to physicians not asking about CAM (57%), not believing that physicians should know about their CAM use (46%) and due to past (2%) or potential (3%) discouragement by physicians [138].

According to a review by Rowe and Baker, around 40%-70% of US patients did not report their CAM use to their doctors whereas 91% of patients who seek care from naturopathic doctors discussed their use of prescription medication. The same review also mentioned some of the most common reasons for non-disclosure of using herbal supplements such as apprehension of disapproval, misunderstanding, disinterest, or lack of knowledge by their physicians in addition to patients’ assumption that the supplements have no connection to their current care [88, 139-143].

Physicians are expected to educate themselves and their patients about the effectiveness and harmful interactions of herbal preparations. Belief in herbal products' efficacy based on advertising, advice from friends, personal experience, dissatisfaction with the conventional health care system, and a desire to be in control of own life and health are among the most common reasons mentioned by patients for using herbal supplements. Since the subject of herbal medicine is complex, physicians often need to discuss the merits and drawbacks of herbs as part of normal history taking through good communication and open discussion by withholding judgment to reach decision making and “relationship-centered” care [144,145]. Patients have very limited and reliable resources regarding the use of herbal medicine, and their most common sources of advice are friends and relatives [146]. Some overseas studies have also identified that medical students and resident physicians are not in a better position than patients because they lack the relevant knowledge and therefore cannot provide expert advice to patients [147-149]. Hence, many patients and providers are not aware of herb-related complications, adverse reactions, and HDI [87]. Patients should be free to choose what healthcare they want but should be informed about the safety and efficacy of their choice [150]. Interprofessional communication (IPC) between health care practitioners (HCP), services, and patients is also critical in providing safe and effective patient-centered care [151].

**Figure 3:**
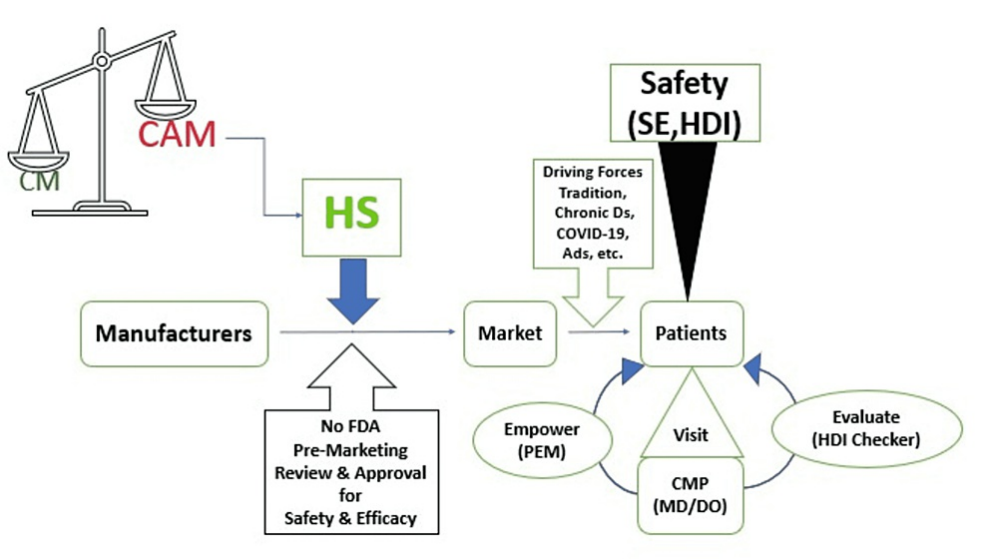
Market access of herbal supplements and physicians’ role in use assessment CM: Conventional Medicine, CAM: Complementary and Alternative Medicine, HS: Herbal Supplement, CMP: Conventional Medicine Practitioner, MD: Doctor of Medicine, DO: Doctor of Osteopathy, PEM: Patient Education Material, HDI: Herb-Drug Interaction, SE: Side Effects, Ds: Diseases, Ads: Advertisements, etc.: Other Driving Forces like Affordability, Social Influence, Dissatisfaction with CM and the likes. Figure Credit: Gashaw Hassen

Physicians can play pivotal roles in promoting patient safety and mitigating the health risks of herbal supplements. Encouraging patients to discuss their use of herbal supplements, addressing any adverse effects including HDI during medication history taking, and providing reliable information about the authenticity, safety, and effectiveness of a disclosed herbal product are very helpful during each encounter with physicians (Figure 3) [95,100,105,152]. Physicians should keep themselves informed by reviewing scientific evidence available in literature databases like PubMed, publications by professional associations like American Medical Associations (AMA), and government websites like NCCIM and FDA [153-157]. Physicians can also help notify herbal adverse reactions to FDA by filling out a safety report and submitting the complaint through the Safety Reporting Portal [56].

There is a growing tendency to integrate CAM with CM in centers and clinics which are both reimbursed with insurance coverage and have close ties to medical schools and teaching hospitals [158]. Physicians can contribute to the development and delivery of courses integrated with continuing medical education (CME) as well as the curricula of medical schools and graduate medical education (GME) training addressing the clinical implications of herbal supplements including the management of HDI [138,154,159,160-162]. Due to the paucity of evidence concerning the safety and efficacy of herbal supplements in medical practice, physicians can fill the gap by involving themselves in clinical research through collaboration with grant providers like NIH [163,164].

Physicians fostering collaboration with both mainstream and internet media is critical in educating safety of herbal supplements to boost consumers’ awareness [165]. There is a 20-time increase in the number of dietary supplements including herbal products over the last three decades since 1994 of DSHEA which enabled manufacturers to exploit the regulatory loophole. Physicians should be vocal to advocate for stricter regulation of the herbal manufacturing industry. It is also time now for physicians in the US to push for the passing of the Dietary Supplement Listing Act of 2022 which mandates manufacturers to uphold a comprehensive listing of product ingredients along with warnings, precautions, and allergen information [166,167].

Finally, it is worth introducing an exemplary physician who is spearheading the fight to stop herbal products from endangering the lives of unsuspecting consumers, Dr. Stephen Barrett. He is a retired psychiatrist from North Carolina who is an award-winning health educator, author, editor, peer-reviewer, and consumer advocate. His renowned contributions are reflected through the multiple websites he is operating including quackwatch.com. Currently, he is co-editing ‘consumer health digest’ which is a free weekly newsletter summarizing scientific reports, legislative developments, enforcement actions, news reports, website evaluations, recommended and non-recommended books, and other information relevant to consumer protection and consumer decision-making [168].

The following table (Table 12) summarizes key roles and major activities physicians can contribute to the overall effort to minimize the health risks of herbal supplements in clinical practice.

**Table 12:** Major roles of physicians in safeguarding herbal supplement users HS: Herbal Supplement, App: Application, PEM: Patient Education Material, HDI: Herb-Drug Interaction, FDA: Food and Drug Administration, NIH: National Institutes of Health, NCCIM: National Center for Complementary and Integrative Health, CME: Continuing Medical Education, MSRF: Medical Student, Resident, and Fellow, ADR: Adverse Drug Reaction.

Key Roles	Major Activities	Resources
Patient Care	Discuss the use of HS	HerbLlist App, Monographs, PEM (Appendix/Figure 4)
	Review medication history	HDI Checker tools (Table 8)
	Determine the contents of the HS the patients are taking	Dietary Supplement Label Database
ADR Reporting	Notify FDA of HS adverse outcomes identified in clinical practice	FDA’s Safety Reporting Portal
Education	Develop and deliver clinically relevant courses related to HS safety	CME, MSRF training platforms
Clinical Research	Involve in research activities aiming at evidence-based medical practice	NCCIM, NIH
Media	Educate the safety and health risks of HS	Mainstream and Social Media Outlets
Regulation	Advocate for stricter control of the quality and safety of HS	Local, State, and Federal Legislators

## Conclusions

Herbal supplement usage is complex and interwoven into anecdotes, cultural traditions, social influence, health misinformation, affordability, accessibility, profitable industry, sophisticated marketing, and consumer autonomy. Herbal medicine is practiced everywhere irrespective of geographic and demographic variations. Still little is known about the safety and efficacy of most herbal products. DSHEA of 1994 severely limited the regulatory ability of the FDA. Patients tend to not disclose or underreport the use of herbal supplements to their physicians. Since more patients are dissatisfied with the herb-related knowledge of conventional medical practitioners, it is advised to be a well-informed physician who stayed abreast with the current trends in order to address herb-related questions. It is not advisable to generalize about the efficacy and safety of herbal supplements. Government websites, NIH’s HerbList App, and HDI checkers are reliable and neutral sources of information. Physicians can enormously contribute to the overall safety of herbal supplements by collaborating with educational and research organizations, media outlets, and lawmakers. Further clinical trials, formal training, regulatory amendment, and global collaboration are crucial in curbing the health care burden of herbal supplements in the US clinical practice.

## Figures and Tables

**Figure 4:**
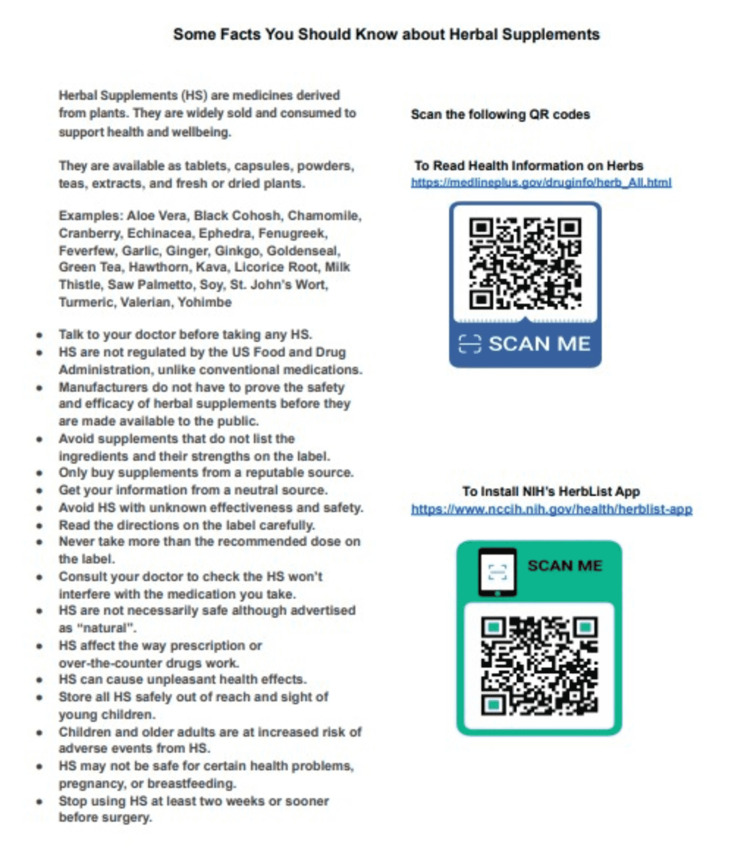
Sample patient education material about safety of herbal supplements Figure Credit: Gashaw Hassen
